# Catalysis-Hub.org, an open electronic structure database for surface reactions

**DOI:** 10.1038/s41597-019-0081-y

**Published:** 2019-05-28

**Authors:** Kirsten T. Winther, Max J. Hoffmann, Jacob R. Boes, Osman Mamun, Michal Bajdich, Thomas Bligaard

**Affiliations:** 1 SUNCAT Center for Interface Science and Catalysis, SLAC National Accelerator Laboratory, 2575 Sand Hill Road, Menlo Park, California, 94025 United States; 2 SUNCAT Center for Interface Science and Catalysis, Department of Chemical Engineering, Stanford University, Stanford, California 94305 United States

**Keywords:** Research data, Materials for energy and catalysis, Cheminformatics, Computational chemistry

## Abstract

We present a new open repository for chemical reactions on catalytic surfaces, available at https://www.catalysis-hub.org. The featured database for surface reactions contains more than 100,000 chemisorption and reaction energies obtained from electronic structure calculations, and is continuously being updated with new datasets. In addition to providing quantum-mechanical results for a broad range of reactions and surfaces from different publications, the database features a systematic, large-scale study of chemical adsorption and hydrogenation on bimetallic alloy surfaces. The database contains reaction specific information, such as the surface composition and reaction energy for each reaction, as well as the surface geometries and calculational parameters, essential for data reproducibility. By providing direct access via the web-interface as well as a Python API, we seek to accelerate the discovery of catalytic materials for sustainable energy applications by enabling researchers to efficiently use the data as a basis for new calculations and model generation.

## Introduction

Electronic structure methods based on density functional theory (DFT) hold the promise to enable a deeper understanding of reaction mechanisms and reactivity trends for surface catalyzed chemical and electrochemical processes and eventually to accelerate discovery of new catalysts. As the access to large-scale supercomputer resources continue to increase, the generated data from electronic structure calculations is also expected to increase^1^. This leads us to a new paradigm of computational catalysis research where the increasing amount of computational data can be utilized to train surrogate models to direct and accelerate efforts for the identification of improved catalysts. Through collaborative efforts and the development of open-source databases and software tools, there is a great prospect for automated catalyst design and discovery^2^.

In the regime of data-driven catalysis research, it is important that data can be accessed efficiently and selectively so that meaningful subsets can be leveraged to make new computational insights into catalyst design. Therefore, development of advanced approaches for storing and accessing relevant data, such as the establishment of curated open access databases is critical^3^. Ensuring that data is findable, accessible, inter-operational, and reusable, in correspondence with the FAIR guiding principles for data management^4^, is an important step towards making data machine as well as human readable.

Several databases for electronic structure calculations have emerged in the last decade with great success, such as Materials Project^5^, Open Quantum Materials Database (OQMD)^6^, the Novel Materials Discovery (NoMaD) repository^7^, Automatic Flow for materials discovery (AFLOW)^8^, the ioChem-BD platform^9^ and the Computational Materials Repository (CMR)^10–12^. While the databases mentioned above primarily feature calculations for crystal strucutres, 2D materials and/or gas phase molecules, the representation of specialized properties such as catalytic activity introduces additional complexity to the database design. A proper representation requires a specific database structure, where reaction energies, chemical species, surface facets, and surface compositions have been parsed, by tying together the output of several calculations.

A database for chemical reactions on surfaces was previously achieved by CatApp^13^, where reaction and activation energies for approximatly 3,000 reactions on primarily closed-packed transition metal surfaces are accessible from a web browser. However, since CatApp does not store the atomic structures or the detailed computational settings and output of the electronic structure calculations, data reproducibility is limited. Also, atomic structures are essential for constructing high-quality models of catalytic activity since the catalytic properties of a surface are determined by the local atomic structure of the active site.

Here, we present a specialized database for reaction and activation energies for chemical reactions on catalytic surfaces which includes electronic structure geometries and contains more than 100,000 adsorption and reaction energies. The database is available from the web platform https://www.catalysis-hub.org that serves as a framework for sharing data as well as computational tools for catalysis research. The platform features several other applications (apps) for plotting results, creating and analyzing calculations, setting up new surface and adsorbate geometries^14^ and making machine learning predictions for adsorption energies^15,16^. A full description of the platform is beyond the scope of this work which will focus on the Surface Reactions database.

## The Surface Reactions Database

The Surface Reactions database stores adsorption, reaction, and reaction barrier energies, obtained from electronic structure calculations, for processes occurring on catalytic surfaces. The main goal of the platform is to make these results easily available to the public and other researchers to facilitate new catalyst discoveries. By enabling researchers to upload their own results to the platform, we seek to further enhance data sharing. We are particularly interested in chemical reactions of relevance for sustainable energy applications, such as conversion of CO_2_ and synthetic gas to fuels^17,18^, electrochemical fuel cells^19,20^, and production of fuels and chemicals from electrochemical approaches^21^. The catalytic materials of interest for these applications includes transition metals and alloys, metal-oxides and oxy-hydroxides, perovskites, layered 2D materials, and metal-chalcogenides.

**Fig. 1:**
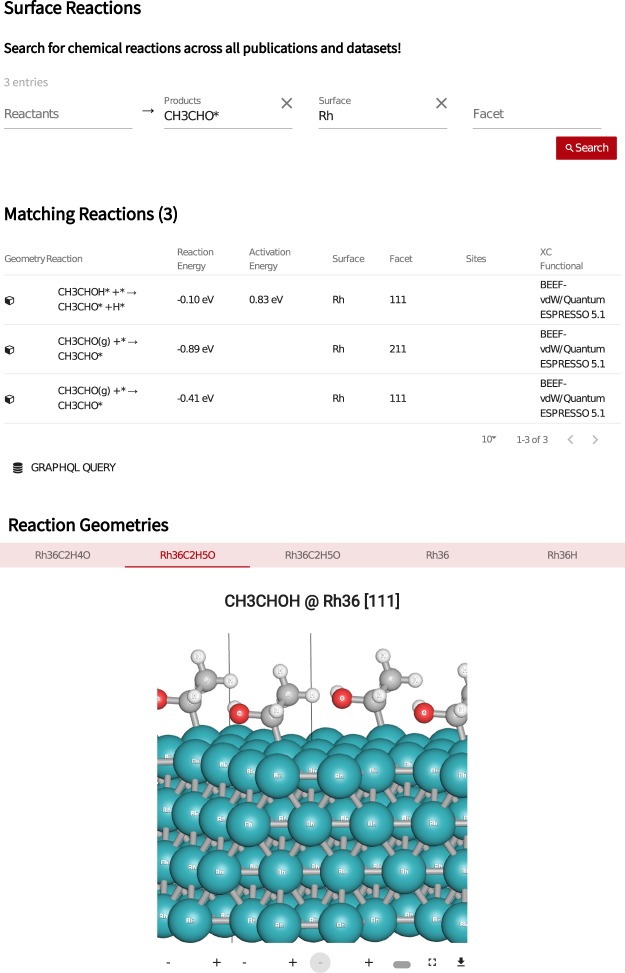
Web interface to the Surface Reactions database, where users can search for reactions by choosing reactants, products, surface composition and/or surface facet. When selecting a reaction, atomic geometries can be visualized for all DFT calculations involved.

In order to model heterogeneous catalytic systems from electronic structure theory, researchers generally use simplified surface slab structures (see example in Fig. 1) to approximate catalyst surfaces, where different adsorption and active sites are sampled in order to generalize the model to more realistic conditions, such as catalytic nanoparticles^22^. The calculation of a reaction energy typically involves at least three electronic structure calculations, including the clean surface slab, the surface with the adsorbed species, and gas phase references of the adsorbate. Also, prior to calculating adsorption energies, the structure of the surface slab is optimized starting from a bulk calculation, just an additional calculations are necessary in order to obtain the transition state geometry that determines the activation barrier for a reaction. We handle this complexity by storing all the atomic geometries for the calculations involved, including the bulk structure if available, and linking the structures to our collection of pre-parsed reaction and activation energies. With this approach, we are ensuring the reproducibility of reaction energies, by mapping the compiled results to the each individual DFT calculation.

In the Surface Reactions app at, https://www.catalysis-hub.org/energies, the user can search for chemical reactions by specifying reactants, products, surface composition, and/or surface facet. The result of the search will be returned as a list of rows in the browser showing the surface composition, the chemical equation of the reaction, reaction energy, activation energy (when present), and adsorption sites. Selecting the geometry symbol to the left of a given row will expand the result, allowing users to browse the atomic structures linked to the reaction and see publication info and calculational details, including the total DFT energy obtained, DFT code, exchange correlation functional and eventual energy corrections. Additional calculation details can be accessed at the web API at http://api.catalysis-hub.org/graphql, where a link is provided for each structure shown in the browser. An example of a reaction search is given in Fig. 1, showing the results for reactions taking place on Rhodium surfaces that contains CH_3_CHO* on the right hand (product) side of the chemical equation. The five atomic structures involved in the reaction can be spatially repeated in the browser for better visualization and downloaded in a several formats including CIF, JSON, xyz, VASP POSCAR, CASTEP Cell and Quantum Espresso input.

### Featured datasets

The database contains results from more than 50 publications and datasets available at https://www.catalysis-hub.org/publications, where reactions can be browsed publication-wise together with visualization of atomic geometries. Most of the datasets stem from already published work and contain a direct link to the publication homepage via the digital object identifier (DOI). A collection of to be published/recently submitted datasets are also made available. Recently uploaded datasets includes studies of syngas to C+ Oxygenates conversion on transition metals^18^, oxygen reduction and hydrogen oxidation on metal-doped 2D materials^20^, solvated protons at the electrochemical water-metal interface^23^, single-atom catalysts for the oxygen reduction reaction^24^, and a large scaly study of chemical adsorption on bimetallic alloy surfaces^25^.

**Fig. 2:**
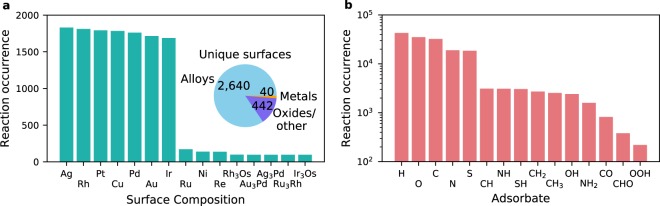
Overview of the contents of the Surface Reactions database. (**a**) Fifteen most occurring surface compositions for the reactions. Although pure, noble metals are most prevalent when counting by unique surface composition, the database is overall dominated by a large diversity of metallic alloys and oxides. (**b**) Fifteen most prevalent adsorbates taking part in reactions, with occurrence shown on a log scale.

The database contains roughly 700 different chemical reactions, involving more that 100 adsorbed species and 3,000 different catalytic material surfaces, where the fifteen most prevalent surface compositions and chemical species are shown in Fig. 2a,b respectively. When considering unique surface composition, the most prevalent materials are the pure, noble metals such as Ag, Rh, Pt and Cu which are well-known as good catalysts. However, as a whole, the database contains a large variety of alloy surfaces and oxides, serving as candidates for cheaper and more abundant catalytic materials. With regards to chemical species, the database has a large collection of mono-atomic adsorbates H, O, C, N and S, while hydrogenated species are an order of magnitude lower in occurrence.

**Fig. 3:**
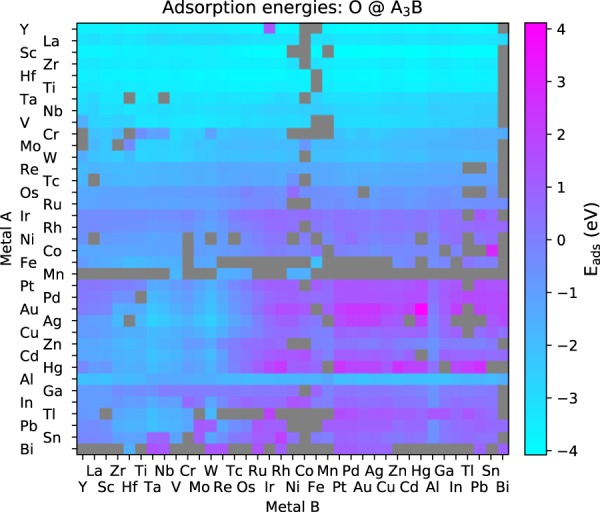
Adsorption energies of atomic oxygen (O) adsorbed onto L1_2_ bimetallic alloys with a A_3_B composition. The adsorption energy corresponds to the reaction: H_2_O(g) - H_2_(g) + * → O*, with O adsorbed to the most stable site obtained. From ref.^25^.

A large part of the reaction energies stem from new high-throughput study of chemical adsorption and hydrogenation on more than 2,000 bimetallic alloy and pure metal surfaces^25^ available at https://www.catalysis-hub.org/publications/MamunHighT2019 as well as from the Materials Cloud archive^26^. As an example, Fig. 3 shows the adsorption energies of atomic oxygen (O) on the subset of alloys with A_3_B composition in the L1_2_ structure, where A and B are chosen among 37 metals. The adsorption energies are plotted as a function of both metal A and B, that are arranged on an improved Pettifor scale^27,28^, which gives rise to a smooth variation of the adsorption energies with composition (a small rearrangement was applied for the magnetic elements Ni, Co, Fe and Mn). The sampled surfaces are seen to cover an extensive range in adsorption energies, spanning more that 5 eV with strong adsorption (low values) for early transition metal alloys (top left corner) and weak adsorption (high values) for noble and late metals (lower right corner). A link to the script used to plot by fetching the data directly with a Python API is provided in the *Code* *Availability* section. We refer to^25^ for the computational details of this study.

Since the database contains entries with different DFT codes and exchange-correlation functionals, reaction energies from different datasets are not necessarily directly comparable, even though trends within a dataset are well-converged. Thus, care should be taken when making quantitative studies that combines reaction energies from different publications. The database predominantly consist of calculations performed with Quantum Espresso^29^, VASP^30,31^ and GPAW^32^. Most prevalent exchange-correlation functionals used are BEEF-vdW^33^ which have shown to have superior performance for adsorption^34^ as well as transition state energetics^35^, RPBE^36^ which improves the PBE adsorption energy for purely chemisorbed systems^34^, and PBE + U^37,38^ which is well-suited to describe transition metal oxide surfaces. Since the Surface Reactions database, as a minimum, tracks the DFT code and functional, datasets with similar calculation settings can still be identified and combined. We note that structure specification such as lattice constants, adsorption sites, and the number of atomic layers in the surface slab can also impact the calculated reaction energetics, just as calculation settings such as the plane-wave energy cutoff, k-point sampling and U-values can affect the result.

### Data accessibility

**Fig. 4:**
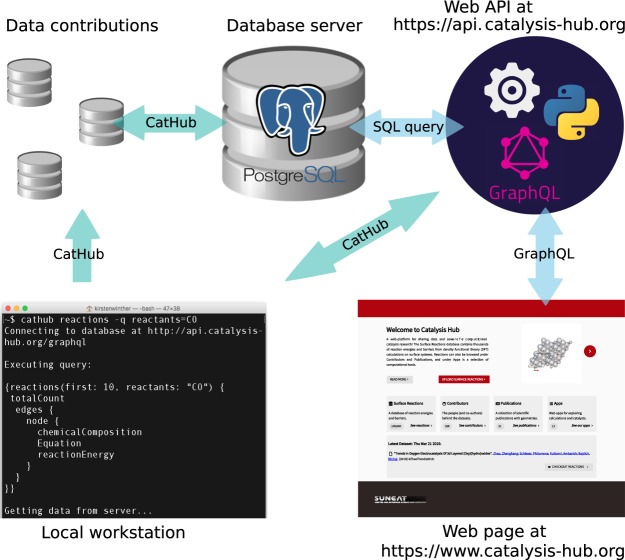
Schematic overview of the database platform, showing the relation between the database server, the backend and the frontend applications.

An overview of the infrastructure of the database is shown in Fig. 4. The platform consists of a database server where the data is stored, a web application programming interface (API) that handles queries to the database, and a frontend application which serves the main web page. Data fetching from the backend to the main web page is handled by a graph based query language, GraphQL (https://graphql.org/), whereas a a Python API is provided by the CatHub software module, which is available within the Zenodo Repository^39^.

Data is stored in a relational database instance, where structured tables with reaction and publication information enables fast sub-selections of data. The atomic structures are stored in ASE database layout, where ASE^40^ is a popular software package for setting up and managing atomic structures, with interfaces to a large set of popular electronic structure codes. The ASE database is developed specifically for storing atomic structures, computational results and parameters, making it a natural choice for handling the atomic structures of reaction intermediates. An overview of the structured query language (SQL) layout of the database is provided in the *Methods* section.

The CatHub software package provides an additional interface to the database, that can be used for data fetching directly from a Python script or the terminal. In practice, the data is fetched by sending a GraphQL query to the database backend as a HTTP request, which returns a JSON dictionary with the selected data (see Fig. 6 for an example of a graphQL query). A code snippet with an example of how to obtain reaction energies in Python is shown below,

from cathub.query import get_reactions

get_reactions (n_results=10,

chemical Composition=‘~Ni’,

reactants=‘CO2’)

which will return the first ten reactions involving carbon dioxide on the reactants side, on surfaces containing Nickel.

The CatHub module also provides a Command Line Interface (CLI) to be used from the terminal. For example, a wrapper around the ASE database CLI allows users to access the atomic structures in the database. The query below will select all atomic structures from the database containing both Silver and Strontium without any restriction on stoichiometric ratio,

cathub ase AgSr --gui

returned as list with atomic structure and calculational details, including the total potential energy, forces and magnetic moments. The –gui option will open the selected atomic structures directly in the ASE GUI visualizer.

Another core feature of the CatHub software is to aide the submission of new datasets to the platform by organizing a given folder of output files into a structure suitable for uploading. With this feature we seek to facilitate data exchange and promote publications of the catalysis and surface science communities. Contributing is open to everyone, where any self-contained dataset (gas phase references, empty slab, adsorbate geometry) of ASE readable DFT output files is welcome. Instructions for how to upload data are available from https://www.catalysis-hub.org/upload.

## Discussion

We believe that the Surface Reactions database will be of great benefit to the scientific community and will aid researchers in their search for new materials for catalysis and sustainable energy applications. By creating a platform for sharing recent scientific results we are enabling community members to efficiently build on top of each other’s work with direct access to the computational data from several channels. To these ends, community contributions are strongly encouraged.

We wish to ensure that the database has both substantial breadth as well as depth; i.e. covering a large range of different materials and reactions. An increased diversity of data is accomplished by featuring data from a large number of publications. This is demonstrated through the many small and focused datasets which have already been uploaded. This also ensures that the database contains catalytic materials from recent cutting-edge research which will be further facilitated by contributions from a diversity of research groups. On the other hand, the generation of surrogate modes, such as machine learning algorithms, generally require vast amount of systematic generated data. Therefore, the database also contains large computationally-consistent datasets targeted for machine learning purposes, such as the bimetallic alloys dataset. In this regard, we are seeking to populate the database with other large-scale datasets in the future.

One concern regarding the breadth and depth of data is how to obtain reliable reaction energy barriers for a large set of reactions and materials. Since the energy barriers determine the kinetics (or reaction rate) of a chemical reaction, a good prediction is important for getting a quantitative measure for the catalytic activity and selectivity. Due to the high computational cost of determining the transition state of energy barriers, only a fraction of the reactions in the database have an associated energy barrier calculated from DFT. Instead, our focus has been on populating the database with a large set of adsorption energies, which are significantly cheaper to compute and can serve as descriptors to model reaction energies and barriers^41^. In time, advanced machine learning techniques to speed up energy barrier calculations^42^, and targeted kinetic systems of interest will supply more accurate barrier energetics to the existing data. These can serve as input to microkinetic models to obtain reaction rate predictions for a large collection of reactions and surfaces^43^.

Moving forward, integrating Catalysis-Hub with automated workflows for computational catalysis, will enable a systematic expansion of the Surface Reaction database. Such an implementation will ensure full tractability of calculation methods, software and parameters used for calculations, further improving the reproducibility and reusability of the data. Furthermore, linking catalysis-hub to other electronic structure databases, and conforming to semantic web standards for data interchange^44,45^, will improve the machine-readability and FAIRness^4^ of the data. In this regard, the development of a vocabulary, or *ontology*, suitable for heterogeneous catalysis and electrochemistry, will be beneficial for a meaningful metadata labeling of reactions with respect to structural parameters, such as adsorption site - and orientation. Well established ontologies including the Crystallographic Information Framework (CIF)^46,47^ and the IUPAC International Chemical Identifier (InChI)^48^, exists for crystals and chemicals, respectively, and recently an international chemical identifier for reactions (RInChI), was proposed^49^. Bridging these with an ontology for adsorbate-surface geometries, based on graph-theory approaches^14^, will be a first step for developing a ontology for heterogeneous catalysis.

## Methods

This section provides a description of the database structure as well as the frontend and backend applications that underlies the web interface.

**Table 1:** SQL table structure for the Surface Reactions database specific tables.

Table name	Column name	Data type
reactions	id	integer
	chemicalComposition	text
	surfaceComposition	text
	facet	text
	sites	jsonb
	coverages	jsonb
	reactants	jsonb
	products	jsonb
	reactionEnergy	numeric
	activationEnergy	numeric
	dftCode	text
	dftFunctional	text
	username	text
	pubId	text
	textsearch	tsvector
reactionSystems	id	integer
	name	text
	energyCorrection	numeric
	aseId	text
publications	id	integer
	pubId	text
	title	text
	authors	jsonb
	journal	text
	volume	text
	number	text
	pages	text
	year	smallint
	publisher	text
	doi	text
	tags	jsonb
	pubtextsearch	tsvector
publicationSystem	aseId	text
	pubId	text

**Table 2:** PostgreSQL table structure of the systems table of the ASE database, listing column names and datatypes

Column name	Data type
id	integer
uniqueId	text
ctime	double precision
mtime	double precision
username	text
numbers	integer[]
positions	double precision[][]
cell	double precision[][]
pbc	integer
initialMagmoms	double precision[]
initialCharges	double precision[]
masses	double precision[]
tags	integer[]
momenta	double precision[]
constraints	text
calculator	text
calculatorParameters	jsonb
energy	double precision
freeEnergy	double precision
forces	double precision[][]
stress	double precision[]
dipole	double precision[]
magmoms	double precision[]
magmom	double precision
charges	double precision[]
keyValuePairs	jsonb
data	jsonb
natoms	integer
fmax	double precision
smax	double precision
volume	double precision
mass	double precision
charge	double precision

Array datatypes are marked with “[]” for 1D arrays and “[][]” for 2D arrays. The JSONB datatype saves dictionaries in a binary format that is fast to process and allows for fast queries on key value pairs and calculational parameters.

### Data structure

**Fig. 5:**
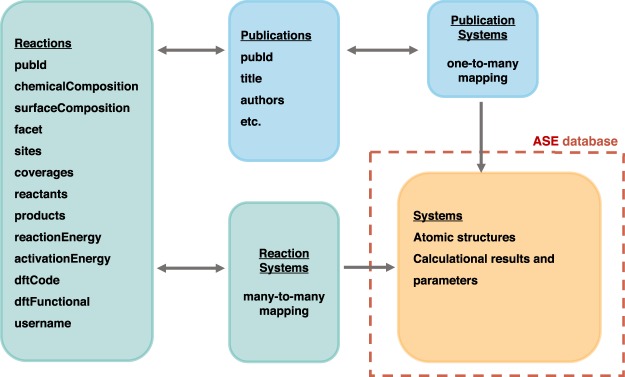
Database table layout.

Data is stored on a PostgreSQL (https://www.postgresql.org/) database instance on Amazon Web Services where it is backed up continuously. Using structured query language (SQL), data is stored in a collection of ordered tables, and selections on properties (columns) can be applied to return a subset of rows and columns from the tables. A schematic overview of the SQL tables used for the Surface Reactions database is shown in Fig. 5. Separate tables are used to store publications, reactions, and atomic structures (systems), allowing for one-to-many and many-to-many mappings between these properties. The Reactions table contains reaction specific info, so that fast queries on chemical composition of the surface, reaction energy, and adsorption sites can be performed. Each reaction is linked to the atomic structures involved (such as adsorbed species, empty slabs, gas phase references, and bulk structure) in the systems table. Also, both reactions and atomic structures are linked to the corresponding entry in the publication table.

The full layout of the SQL tables is given in Tables 1 and 2, listing the columns and datatypes of the Surface Reactions database specific tables and the ASE database systems table, respectively. The Systems table of the ASE database contains information about the geometry (such as atomic numbers, positions, and constraints), calculator settings, and the output of the calculation (such as energy, forces, and magnetic moments). An update of the ASE database in connection to this project enables us to utilize native ARRAY and JSONB datatypes for PostgreSQL v-9.4+. The JSONB datatype is a binary JSON format that stores user-defined keys and values in a search-optimized way, which enables faster queries on user defined key-value-pairs. This ensures that a larger amount of user-defined metadata can be assigned to each atomic structure at a low cost. The ARRAY data type is used to store arrays such as the atomic positions and numbers, which ensures that selections on the chemical composition (and potentially local atomic structure in the vicinity of adsorbates) can be executed directly in SQL.

### Frontend and backend applications

The main web page is served by a frontend application^50^ that runs on a Node.js instance on the Heroku Cloud Application Platform. The frontend source code is implemented using the React framework. Atomic structures are visualized in the browser using the ChemDoodle^51^ web component.

Retrieval of data from the database server is managed by a backend application^52^ which is a collection of software that runs on a Python framework on Heroku Cloud Application Platform. The backend is build with Flask (https://pypi.org/project/Flask), a microframework for web development in Python, and uses the Python SQL toolkit SQLAlchemy (https://www.sqlalchemy.org/), for connecting to the database server and handling relations (such as foreign key constraints and many-to-many mappings) between SQL tables.

**Fig. 6:**
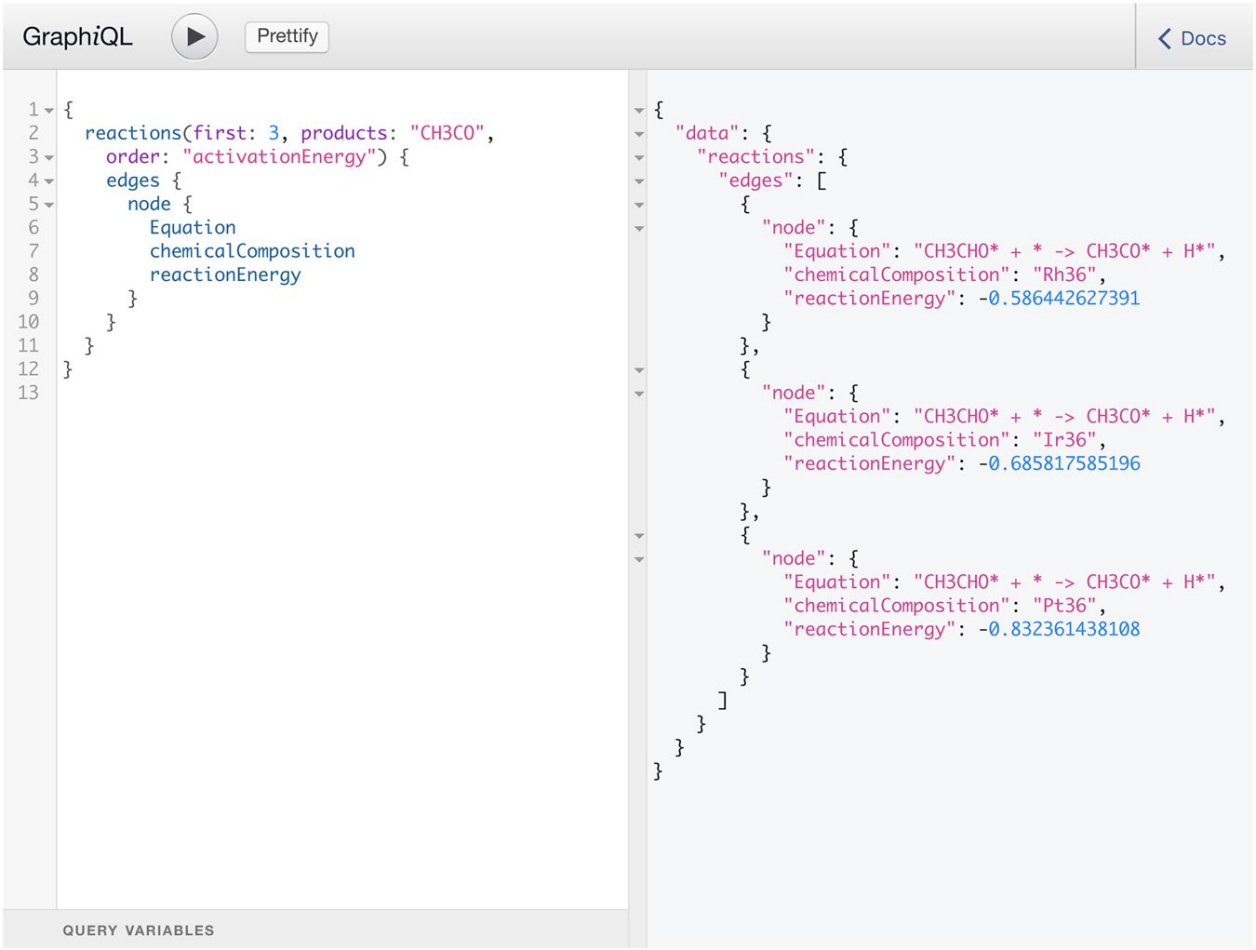
Example of a GraphQL query for reactions, executed in the API web interface. The web API can be accessed at https://api.catalysis-hub.org/graphql.

Data fetching from the backend to the frontend is handled with GraphQL, a graph based query language developed by Facebook as an alternative to representational state transfer (REST). It provides simple and user friendly data-fetching, where the request is sent as a string in JSON-like format that specifies the data to be selectedand a JSON object with the same data structure as the request is returned. The backend can be accessed at https://api.catalysis-hub.org/graphql, where GraphQL queries can be typed directly into the browser. An example of such a query is given in Fig. 6, where the first three reactions involving CH_3_CO on the right hand side, in the order of increasing activation energy, is returned.

## Data Availability

All datasets discussed this study are available from the Surface Reactions database of Catalysis-Hub at http://www.catalysis-hub.org/publications/. In addition, the Bimetallic Alloys dataset^25^, is made available from the Materials Cloud archive^26^. Also, datasets are featured in Google Dataset Search at https://toolbox.google.com/datasetsearch, which will link to the catalysis-hub website.

## Code Availability

All code developed for the Catalysis-Hub platform is made available open source from the SUNCAT Center’s GitHub repository at https://github.com/SUNCAT-Center, which includes the database backend^52^, frontend^50^ and the CatHub python API^39^. The Python script used for plotting the data shown in Fig. 3, using the CatHub API, is made available as a tutorial at https://github.com/SUNCAT-Center/CatHub/tree/master/tutorials/1_bimetallic_alloys/heatmaps.py.
